# Supplementary material for: Clinical Characterization of Targetable Mutations (BRAF V600E and KRAS G12C) in Advanced Colorectal Cancer—A Nation-Wide Study
Source: Int J Mol Sci. 2023 May 22;24(10):9073. doi: 10.3390/ijms24109073 (PMC10219350; doi:10.3390/ijms24109073)
Supplement: Supplementary file 1 [file ijms-24-09073-s001.zip › ijms-2388562-supplementary.pdf]

International Journal of *Molecular Sciences*

MDPI

Table S1. Complete population characteristics. 1

Parameter		Group			
		Overall (N=7604)	BRAF V600E (N=515)	KRAS G12C (N=237)	other KRAS (N=3591)
**Sex**	Female	3132 (41,19%)	295 (57,28%)	101 (42,62%)	1527 (42,52%)
	Male	4471 (58,81%)	220 (42,72%)	136 (57,38%)	2064 (57,48%)
Age [years]	< 30	21 (0,28%)	0 (0,00%)	0 (0,00%)	10 (0,28%)
	31-40	115 (1,51%)	6 (1,17%)	6 (2,53%)	49 (1,36%)
	41-50	432 (5,68%)	22 (4,27%)	13 (5,49%)	190 (5,29%)
	51-60	1298 (17,07%)	74 (14,37%)	36 (15,19%)	588 (16,37%)
	61-70	3192 (41,98%)	222 (43,11%)	111 (46,84%)	1486 (41,38%)
	71-80	2173 (28,58%)	156 (30,29%)	64 (27,00%)	1070 (29,80%)
	over 80	373 (4,91%)	35 (6,80%)	7 (2,95%)	198 (5,51%)
Tumour stage	T1	39 (0,98%)	0 (0,00%)	1 (0,83%)	25 (1,29%)
	T2	291 (7,29%)	10 (3,33%)	11 (9,09%)	144 (7,44%)
	T3	2611 (65,37%)	175 (58,33%)	81 (66,94%)	1243 (64,20%)
	T4	1039 (26,01%)	115 (38,33%)	28 (23,14%)	516 (26,65%)
	Tis	14 (0,35%)	0 (0,00%)	0 (0,00%)	8 (0,41%)
Nodal stage	N0	1006 (25,42%)	57 (19,32%)	37 (30,33%)	486 (25,34%)
	N1a	516 (13,04%)	40 (13,56%)	8 (6,56%)	272 (14,18%)
	N1b	735 (18,57%)	43 (14,58%)	27 (22,13%)	354 (18,46%)
	N1c	209 (5,28%)	11 (3,73%)	8 (6,56%)	114 (5,94%)
	N2a	649 (16,4%)	55 (18,64%)	26 (21,31%)	307 (16,01%)
	N2b	728 (18,39%)	82 (27,80%)	13 (10,66%)	336 (17,52%)
	Nx	115 (2,91%)	7 (2,37%)	3 (2,46%)	49 (2,55%)
Nodal stage (0 vs 1 vs 2)	N0	1006 (26,18%)	57 (19,79%)	37 (31,09%)	486 (26,00%)
	N1	1460 (37,99%)	94 (32,64%)	43 (36,13%)	740 (39,59%)
	N2	1377 (35,83%)	137 (47,57%)	39 (32,77%)	643 (34,40%)

Nodal stage (positive vs negative)	Negative	1006 (26,18%)	57 (19,79%)	37 (31,09%)	486 (26,00%)
	Positive	2837 (73,82%)	231 (80,21%)	82 (68,91%)	1383 (74,00%)
Histology	Adenocarcinoma NOS	6364 (89,93%)	362 (78.02%)	196 (88.69%)	3108 (88.52%)
	Partially mucinous	632 (8,93%)	89 (58,17%)	23 (56,10%)	382 (56,26%)
	Mucinous	35 (0,49%)	6 (3,92%)	0 (0,00%)	10 (1,47%)
	Cribriform or comedo	32 (0,45%)	2 (1,31%)	2 (4,88%)	9 (1,33%)
	MANEC	14 (0,2%)	5 (3,27%)	0 (0,00%)	2 (0,29%)
Mucous component	No	6555 (86,2%)	385 (74,76%)	200 (84,39%)	2975 (82,85%)
	Yes	1049 (13,8%)	130 (25,24%)	37 (15,61%)	616 (17,15%)
Signet cells presence	No	7187 (94,52%)	474 (92,04%)	223 (94,09%)	3357 (93,48%)
	Yes	417 (5,48%)	41 (7,96%)	14 (5,91%)	234 (6,52%)
Lymphocytic infiltration	No	7518 (98,87%)	510 (99,03%)	237 (100,00%)	3549 (98,83%)
	Yes	86 (1,13%)	5 (0,97%)	0 (0,00%)	42 (1,17%)
Grade	G1	590 (12,14%)	26 (7,60%)	17 (10,90%)	299 (12,88%)
	G2	3509 (72,22%)	181 (52,92%)	122 (78,21%)	1708 (73,59%)
	G3	756 (15,56%)	134 (39,18%)	17 (10,90%)	313 (13,49%)
	G4	4 (0,08%)	1 (0,29%)	0 (0,00%)	1 (0,04%)
Grade (1-2 vs 3-4)	G1-2	4099 (84,36%)	207 (60,53%)	139 (89,10%)	2007 (86,47%)
	G3-4	760 (15,64%)	135 (39,47%)	17 (10,90%)	314 (13,53%)
Angioinvasion	No	816 (33,62%)	41 (21,69%)	26 (30,95%)	434 (36,94%)
	Yes	1611 (66,38%)	148 (78,31%)	58 (69,05%)	741 (63,06%)
Perineural invasion	No	740 (50,96%)	44 (38,94%)	25 (46,30%)	375 (53,19%)
	Yes	712 (49,04%)	69 (61,06%)	29 (53,70%)	330 (46,81%)
Primary tumour localization	Right colon	1570 (24,6%)	293 (69,43%)	35 (17,77%)	784 (25,87%)
	Left colon	1849 (28,97%)	58 (13,74%)	51 (25,89%)	825 (27,22%)
	Rectum	2964 (46,44%)	71 (16,82%)	111 (56,35%)	1422 (46,92%)
Primary sidedness	Left colon or rectum	4827 (75,46%)	129 (30,57%)	163 (82,32%)	2255 (74,20%)

	Right colon	1570 (24,54%)	293 (69,43%)	35 (17,68%)	784 (25,80%)
Primary localization (colon vs rectum)	Colon	4149 (58,33%)	398 (84,86%)	107 (49,08%)	1953 (57,87%)
	Rectum	2964 (41,67%)	71 (15,14%)	111 (50,92%)	1422 (42,13%)
Sample type	Endoscopy	1388 (22,91%)	73 (17,76%)	45 (22,84%)	654 (22,58%)
	Needle	218 (3,6%)	7 (1,70%)	12 (6,09%)	90 (3,11%)
	Surgery	4452 (73,49%)	331 (80,54%)	140 (71,07%)	2152 (74,31%)
Sample source	Primary	4968 (87,88%)	346 (90,58%)	153 (83,61%)	2376 (88,43%)
	Metastatic	685 (12,12%)	36 (9,42%)	30 (16,39%)	311 (11,57%)
Location of metastatic samples	local recurrence	81 (11,82%)	3 (0,79%)	5 (2,73%)	39 (1,45%)
	liver	266 (38,83%)	11 (2,88%)	11 (6,01%)	108 (4,02%)
	lung	73 (10,66%)	3 (0,79%)	1 (0,55%)	42 (1,56%)
	peritoneal	148 (21,61%)	12 (3,14%)	5 (2,73%)	76 (2,83%)
	nodal	31 (4,53%)	3 (0,79%)	2 (1,09%)	10 (0,37%)
	ovarian	33 (4,82%)	2 (0,52%)	1 (0,55%)	10 (0,37%)
	small intestine	26 (3,8%)	2 (0,52%)	2 (1,09%)	12 (0,45%)
	CNS	5 (0,73%)	0 (0%)	2 (1,09%)	2 (0,07%)
	skin/subcutaneous	22 (3,21%)	0 (0%)	1 (0,55%)	12 (0,45%)
Biopsy vs surgery for primary samples	Biopsy	1374 (25,49%)	72 (19,15%)	44 (26,19%)	648 (24,98%)
	Surgery	4016 (74,51%)	304 (80,85%)	124 (73,81%)	1946 (75,02%)
Biopsy vs surgery for metastatic samples	Biopsy	259 (23,27%)	8 (11,94%)	14 (30,43%)	110 (20,83%)
	Surgery	854 (76,73%)	59 (88,06%)	32 (69,57%)	418 (79,17%)
Geographic region	Dolnośląskie	832 (10,98%)	63 (12,28%)	26 (10,97%)	386 (10,79%)
	Kujawsko-pomorskie	40 (0,53%)	1 (0,19%)	3 (1,27%)	18 (0,50%)
	Lubelskie	18 (0,24%)	1 (0,19%)	0 (0,00%)	9 (0,25%)

Lubuskie	300 (3,96%)	16 (3,12%)	13 (5,49%)	141 (3,94%)
Łódzkie	1129 (14,91%)	59 (11,50%)	39 (16,46%)	542 (15,16%)
Małopolskie	843 (11,13%)	64 (12,48%)	36 (15,19%)	400 (11,19%)
Mazowieckie	752 (9,93%)	45 (8,77%)	22 (9,28%)	358 (10,01%)
Opolskie	304 (4,01%)	20 (3,90%)	9 (3,80%)	152 (4,25%)
Podkarpackie	481 (6,35%)	34 (6,63%)	11 (4,64%)	234 (6,54%)
Podlaskie	6 (0,08%)	0 (0,00%)	0 (0,00%)	1 (0,03%)
Pomorskie	406 (5,36%)	30 (5,85%)	10 (4,22%)	190 (5,31%)
Śląskie	1379 (18,21%)	105 (20,47%)	40 (16,88%)	652 (18,23%)
Warmińsko-mazurskie	219 (2,89%)	14 (2,73%)	7 (2,95%)	102 (2,85%)
Wielkopolskie	356 (4,7%)	28 (5,46%)	11 (4,64%)	164 (4,59%)
Zachodniopomorskie	509 (6,72%)	33 (6,43%)	10 (4,22%)	227 (6,35%)

Right colon (proximal) included caecum, ascending colon, hepatic flexure and transverse colon. Left colon (distal) included splenic flexure, descending and sigmoid colon. MANEC – mixed adeno-neuroendocrine cancer.

Table S2. Expanded clinical and pathological factors that affected the rate of *BRAF* V600E mutation.

Trait	Group	BRAF V600E	OR	95% CI		p
**Sex**	Male (N=4471)	220 (4.92%)	1	ref.		
	Female (N=3132)	295 (9.42%)	2.009	1.677	2.408	<0.001
Age [years]	< 40 (N=136)	6 (4.41%)	1	ref.		
	41-50 (N=432)	22 (5.09%)	1.163	0.462	2.929	0.749
	51-60 (N=1298)	74 (5.70%)	1.31	0.559	3.069	0.534
	61-70 (N=3192)	222 (6.95%)	1.62	0.706	3.713	0.255
	71-80 (N=2173)	156 (7.18%)	1.676	0.728	3.86	0.225
	Over 80 (N=373)	35 (9.38%)	2.244	0.922	5.459	0.075
Histology	Adenocarcinoma NOS (N=6364)	362 (5.69%)	1	ref.		
	Partially mucinous (N=632)	89 (14.08%)	2.718	2.121	3.482	<0.001
	Mucinous (N=35)	6 (17.14%)	3.43	1.415	8.315	0.006
	Cribriform or comedo (N=32)	2 (6.25%)	1.105	0.263	4.643	0.891
	MANEC (N=14)	5 (35.71%)	9.211	3.071	27.626	<0.001
Mucous component	No (N=6555)	385 (5.87%)	1	ref.		
	Yes (N=1049)	130 (12.39%)	2.267	1.837	2.798	<0.001
Signet cells presence	No (N=7187)	474 (6.60%)	1	ref.		
	Yes (N=417)	41 (9.83%)	1.544	1.104	2.16	0.011
Lymphocytic infiltration	No (N=7518)	510 (6.78%)	1	ref.		
	Yes (N=86)	5 (5.81%)	0.848	0.342	2.102	0.722
Grade	G1 (N=590)	26 (4.41%)	1	ref.		
	G2 (N=3509)	181 (5.16%)	1.18	0.775	1.797	0.441
	G3 (N=756)	134 (17.72%)	4.673	3.024	7.221	<0.001
	G4 (N=4)	1 (25.00%)	7.231	0.727	71.909	0.091
Grade (1-2 vs 3-4)	G1-2 (N=4099)	207 (5.05%)	1	ref.		
	G3-4 (N=760)	135 (17.76%)	4.061	3.218	5.125	<0.001

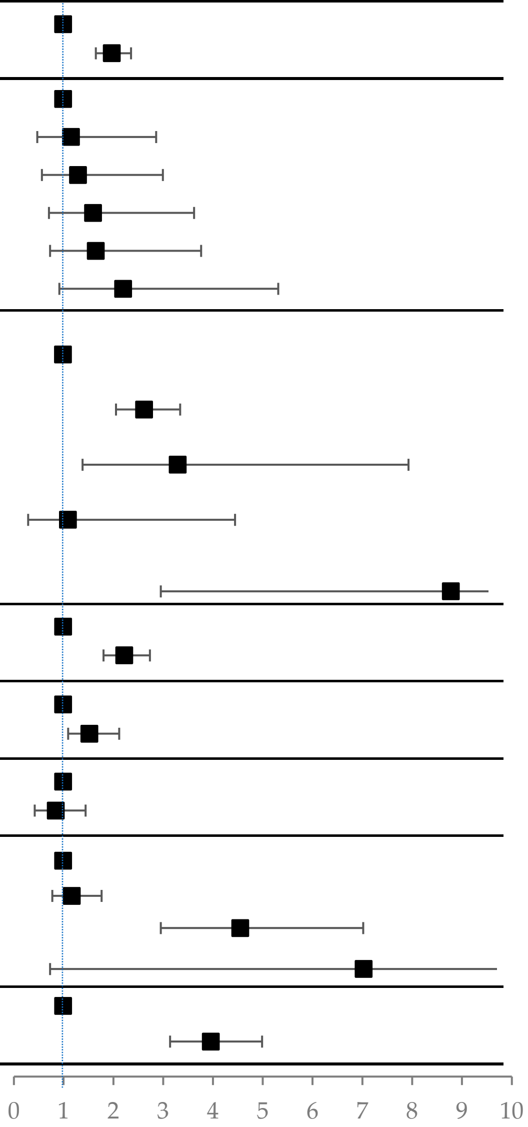

Trait	Group	BRAF V600E	OR	95% CI		p
Primary tumour localization	Rectum/rectosigmoid (N=2964)	71 (2.40%)	1	ref.		
	Left colon (N=1849)	58 (3.14%)	1.32	0.928	1.876	0.122
	Right colon (N=1570)	293 (18.66%)	9.349	7.155	12.216	<0.001
Primary sidedness	Left colon or rectum (N=4827)	129 (2.67%)	1	ref.		
	Right colon (N=1570)	293 (18.66%)	8.356	6.732	10.372	<0.001
Primary localization (colon vs rectum)	Colon (N=4149)	398 (9.59%)	1	ref.		
	Rectum (N=2964)	71 (2.40%)	0.231	0.179	0.299	<0.001
Sample type	Surgery (N=4452)	331 (7.43%)	1	ref.		
	Endoscopy (N=1388)	73 (5.26%)	0.691	0.532	0.897	0.006
	Needle (N=218)	7 (3.21%)	0.413	0.193	0.884	0.023
Tissue source (primary vs metastatic)	Primary (N=4968)	346 (6.96%)	1	ref.		
	Metastatic (N=685)	36 (5.26%)	0.741	0.521	1.055	0.096
Tissue source	Primary (N=4968)	346 (6.96%)	1	ref.		
	Metastatic - other (N=598)	33 (5.52%)	0.78	0.54	1.127	0.186
	Metastatic - local (N=82)	3 (3.66%)	0.507	0.159	1.615	0.251
	Metastatic - CNS (N=5)	0 (0.00%)	--	--	--	--
Biopsy vs surgery for metastatic tissues	Surgery (N=854)	59 (6.91%)	1	ref.		
	Biopsy (N=259)	8 (3.09%)	0.429	0.202	0.911	0.028
Geographic region	Śląskie (N=1379)	105 (7.61%)	1	ref.		
	Łódzkie (N=1129)	59 (5.23%)	0.669	0.481	0.93	0.017
	Małopolskie (N=843)	64 (7.59%)	0.997	0.721	1.377	0.985
	Dolnośląskie (N=832)	63 (7.57%)	0.994	0.718	1.376	0.971

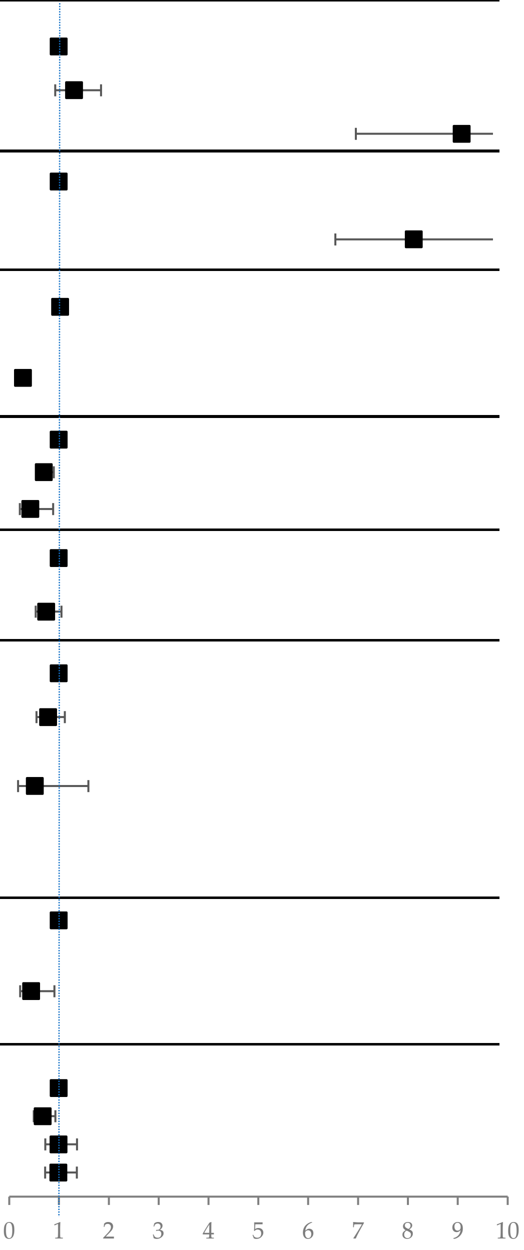

Trait	Group	BRAF V600E	OR	95% CI		p
	Mazowieckie (N=752)	45 (5.98%)	0.772	0.538	1.108	0.161
	Zachodniopomorskie (N=509)	33 (6.48%)	0.841	0.561	1.261	0.403
	Podkarpackie (N=481)	34 (7.07%)	0.923	0.618	1.379	0.695
	Pomorskie (N=406)	30 (7.39%)	0.968	0.635	1.476	0.88
	Wielkopolskie (N=356)	28 (7.87%)	1.036	0.671	1.599	0.874
	Opolskie (N=304)	20 (6.58%)	0.854	0.521	1.402	0.534
	Lubuskie (N=300)	16 (5.33%)	0.684	0.398	1.175	0.169
	Warmińsko-mazur-skie (N=219)	14 (6.39%)	0.829	0.465	1.475	0.523
	Kujawsko-pomorskie (N=40)	1 (2.50%)	0.311	0.042	2.287	0.251
	Lubelskie (N=18)	1 (5.56%)	0.714	0.094	5.416	0.744
	Podlaskie (N=6)	0 (0.00%)	--	--	--	--

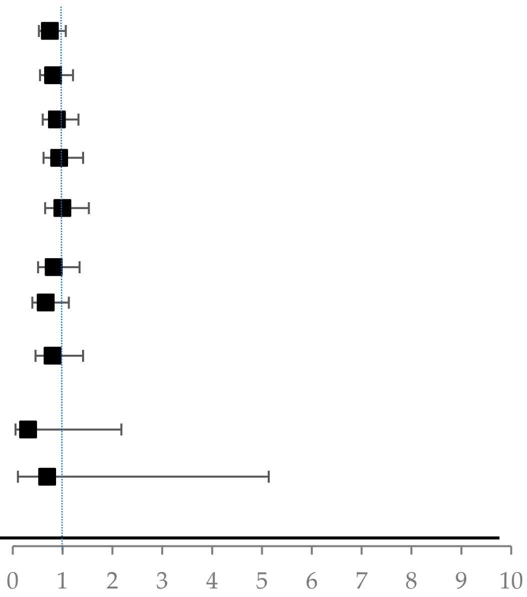

Right colon (proximal) included caecum, ascending colon, hepatic flexure and transverse colon. Left colon (distal) included splenic flexure, descending and sigmoid colon. Rectum included rectosigmoid tumours. MANEC – mixed adeno-neuroendocrine cancer.
p - univariate logistic regressions.

Table S3. Expanded clinical and pathological factors that affected the rate of *KRAS* G12C mutation.

Trait	Group	KRAS G12C	OR	95% CI		p
Sex	Male (N=4471)	136 (3.04%)	1	ref.		
	Female (N=3132)	101 (3.22%)	1.062	0.818	1.38	0.651
Age [years]	< 40 (N=136)	6 (4.41%)	1	ref.		
	41-50 (N=432)	13 (3.01%)	0.672	0.251	1.804	0.43
	51-60 (N=1298)	36 (2.77%)	0.618	0.256	1.494	0.285
	61-70 (N=3192)	111 (3.48%)	0.781	0.337	1.808	0.563
	71-80 (N=2173)	64 (2.95%)	0.657	0.28	1.547	0.337
	Over 80 (N=373)	7 (1.88%)	0.414	0.137	1.256	0.119
Histology	Partially mucinous (N=632)	23 (3.64%)	1	ref.		
	Mucinous (N=35)	0 (0.00%)	--	--	--	--
	Cribriform or comedo (N=32)	2 (6.25%)	1.765	0.398	7.838	0.455
	MANEC (N=14)	0 (0.00%)	--	--	--	--
	Other (N=527)	16 (3.04%)	0.829	0.433	1.586	0.571
Mucous component	No (N=6555)	200 (3.05%)	1	ref.		
	Yes (N=1049)	37 (3.53%)	1.162	0.813	1.66	0.41
Signet cells presence	No (N=7187)	223 (3.10%)	1	ref.		
	Yes (N=417)	14 (3.36%)	1.085	0.626	1.879	0.771
Lymphocytic infiltration	No (N=7518)	237 (3.15%)	1	ref.		
	Yes (N=86)	0 (0.00%)	--	--	--	--
Grade	G1 (N=590)	17 (2.88%)	1	ref.		
	G2 (N=3509)	122 (3.48%)	1.214	0.725	2.032	0.46
	G3 (N=756)	17 (2.25%)	0.775	0.392	1.532	0.464
	G4 (N=4)	0 (0.00%)	--	--	--	--
Grade (1-2 vs 3-4)	G1-2 (N=4099)	139 (3.39%)	1	ref.		
	G3-4 (N=760)	17 (2.24%)	0.652	0.392	1.085	0.1
Primary tumour localization	Rectum (N=2964)	111 (3.74%)	1	ref.		
	Left colon (N=1849)	51 (2.76%)	0.729	0.521	1.021	0.066
	Right colon (N=1570)	35 (2.23%)	0.586	0.399	0.861	0.007

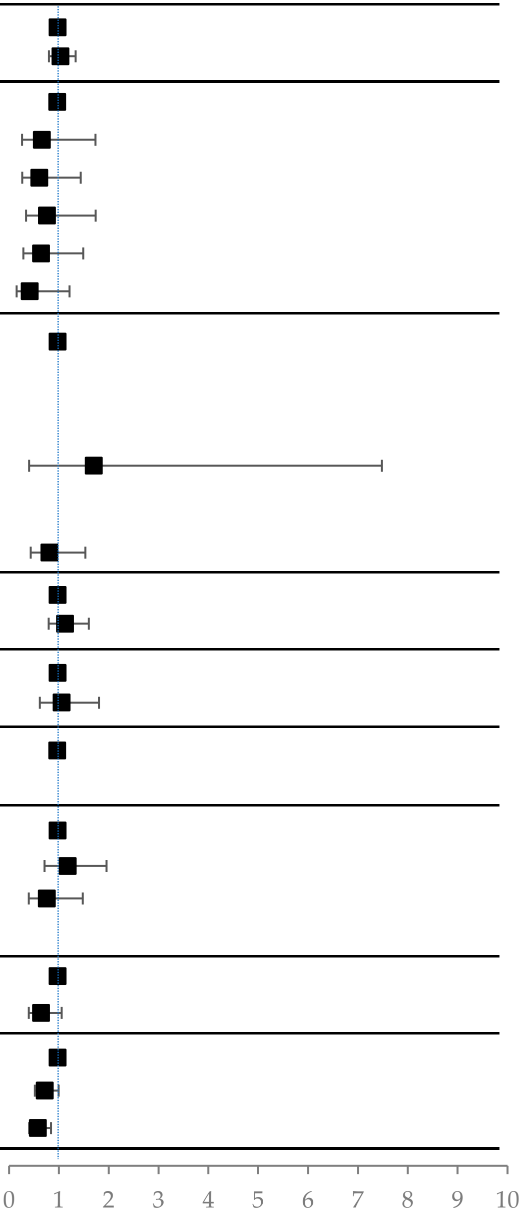

Trait	Group	KRAS G12C	OR	95% CI		p
Primary sidedness	Left colon or rectum (N=4827)	163 (3.38%)	1	ref.		
	Right colon (N=1570)	35 (2.23%)	0.652	0.451	0.944	0.024
Primary localization (colon vs rectum)	Colon (N=4149)	107 (2.58%)	1	ref.		
	Rectum (N=2964)	111 (3.74%)	1.47	1.122	1.925	0.005
Sample type	Surgery (N=4452)	140 (3.14%)	1	ref.		
	Endoscopy (N=1388)	45 (3.24%)	1.032	0.734	1.452	0.856
	Needle (N=218)	12 (5.50%)	1.794	0.979	3.289	0.059
Tissue source (primary vs metastatic)	Primary (N=4968)	153 (3.08%)	1	ref.		
	Metastatic (N=685)	30 (4.38%)	1.441	0.966	2.15	0.073
Tissue source	Primary (N=4968)	153 (3.08%)	1	ref.		
	Metastatic - other (N=598)	23 (3.85%)	1.259	0.805	1.968	0.313
	Metastatic - local (N=82)	5 (6.10%)	2.044	0.815	5.121	0.127
	Metastatic - CNS (N=5)	2 (40.00%)	20.98	3.48	126.47	0.001
Biopsy vs surgery for metastatic tissues	Surgery (N=854)	32 (3.75%)	1	ref.		
	Biopsy (N=259)	14 (5.41%)	1.468	0.771	2.795	0.243
Geographic region	Śląskie (N=1379)	40 (2.90%)	1	ref.		
	Łódzkie (N=1129)	39 (3.45%)	1.198	0.765	1.875	0.43
	Małopolskie (N=843)	36 (4.27%)	1.493	0.944	2.362	0.087
	Dolnośląskie (N=832)	26 (3.12%)	1.08	0.654	1.783	0.764
	Mazowieckie (N=752)	22 (2.93%)	1.009	0.595	1.711	0.974
	Zachodniopomorskie (N=509)	10 (1.96%)	0.671	0.333	1.352	0.264
	Podkarpackie (N=481)	11 (2.29%)	0.783	0.399	1.54	0.479

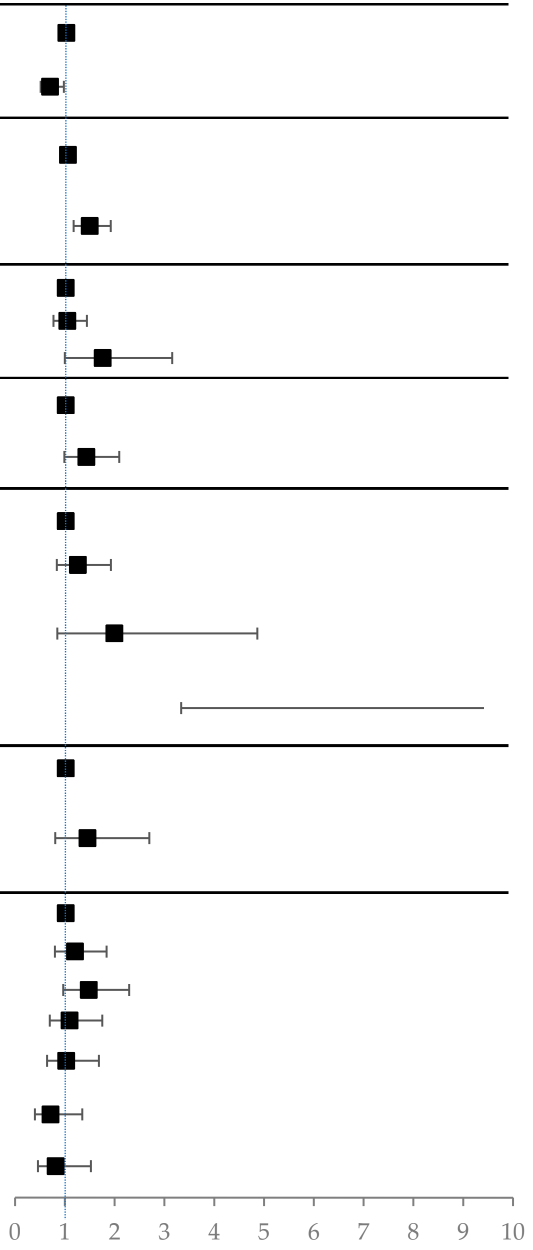

Trait	Group	KRAS G12C	OR	95% CI		p
	Pomorskie (N=406)	10 (2.46%)	0.845	0.419	1.706	0.639
	Wielkopolskie (N=356)	11 (3.09%)	1.067	0.542	2.102	0.851
	Opolskie (N=304)	9 (2.96%)	1.021	0.49	2.128	0.955
	Lubuskie (N=300)	13 (4.33%)	1.516	0.801	2.872	0.201
	Warmińsko-mazur-skie (N=219)	7 (3.20%)	1.105	0.489	2.5	0.81
	Kujawsko-pomorskie (N=40)	3 (7.50%)	2.714	0.803	9.174	0.108
	Lubelskie (N=18)	0 (0.00%)	--	--	--	--
	Podlaskie (N=6)	0 (0.00%)	--	--	--	--

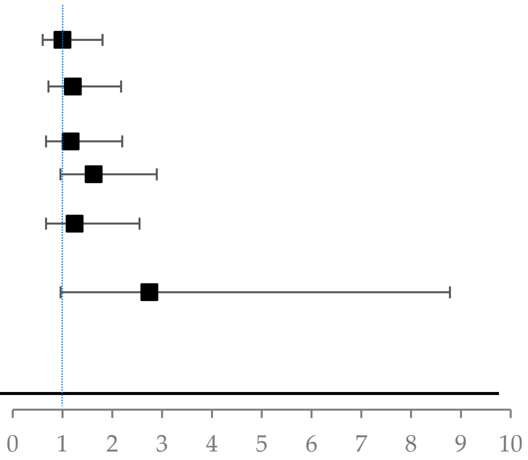

Right colon (proximal) included caecum, ascending colon, hepatic flexure and transverse colon. Left colon (distal) included splenic flexure, descending and sigmoid colon. Rectum included rectosigmoid tumours. MANEC – mixed adeno-neuroendocrine cancer.
p - univariate logistic regressions.

Table S4. Expanded clinical and pathological factors that affected the rate of *KRAS* G12C mutation among other *KRAS* mutations.

Trait	Group	KRAS G12C	OR	95% CI		p
**Sex**	Male (N=2200)	136 (6.18%)	1	ref.		
	Female (N=1628)	101 (6.20%)	1.004	0.77	1.309	0.978
Age [years]	< 40 (N=65)	6 (9.23%)	1	ref.		
	41-50 (N=203)	13 (6.40%)	0.673	0.245	1.848	0.442
	51-60 (N=624)	36 (5.77%)	0.602	0.244	1.488	0.272
	61-70 (N=1597)	111 (6.95%)	0.735	0.31	1.739	0.483
	71-80 (N=1134)	64 (5.64%)	0.588	0.245	1.414	0.236
	Over 80 (N=205)	7 (3.41%)	0.348	0.113	1.074	0.066
Histology	Partially mucinous (N=405)	23 (5.68%)	1	ref.		
	Mucinous (N=10)	0 (0.00%)	--	--	--	--
	Cribriform or comedo (N=11)	2 (18.18%)	3.691	0.753	18.079	0.107
	MANEC (N=2)	0 (0.00%)	--	--	--	--
	Other (N=292)	16 (5.48%)	0.963	0.499	1.856	0.91
Mucous component	No (N=3175)	200 (6.30%)	1	ref.		
	Yes (N=653)	37 (5.67%)	0.893	0.623	1.282	0.541
Signet cells presence	No (N=3580)	223 (6.23%)	1	ref.		
	Yes (N=248)	14 (5.65%)	0.901	0.517	1.571	0.712
Lymphocytic infiltration	No (N=3786)	237 (6.26%)	1	ref.		
	Yes (N=42)	0 (0.00%)	--	--	--	--
Grade	G1 (N=316)	17 (5.38%)	1	ref.		
	G2 (N=1830)	122 (6.67%)	1.256	0.745	2.118	0.392
	G3 (N=330)	17 (5.15%)	0.955	0.479	1.906	0.897
	G4 (N=1)	0 (0.00%)	--	--	--	--
Grade (1-2 vs 3-4)	G1-2 (N=2146)	139 (6.48%)	1	ref.		
	G3-4 (N=331)	17 (5.14%)	0.782	0.466	1.312	0.351
Primary tumour localization	Rectum (N=1533)	111 (7.24%)	1	ref.		
	Left colon (N=876)	51 (5.82%)	0.792	0.562	1.115	0.182
	Right colon (N=819)	35 (4.27%)	0.572	0.387	0.845	0.005

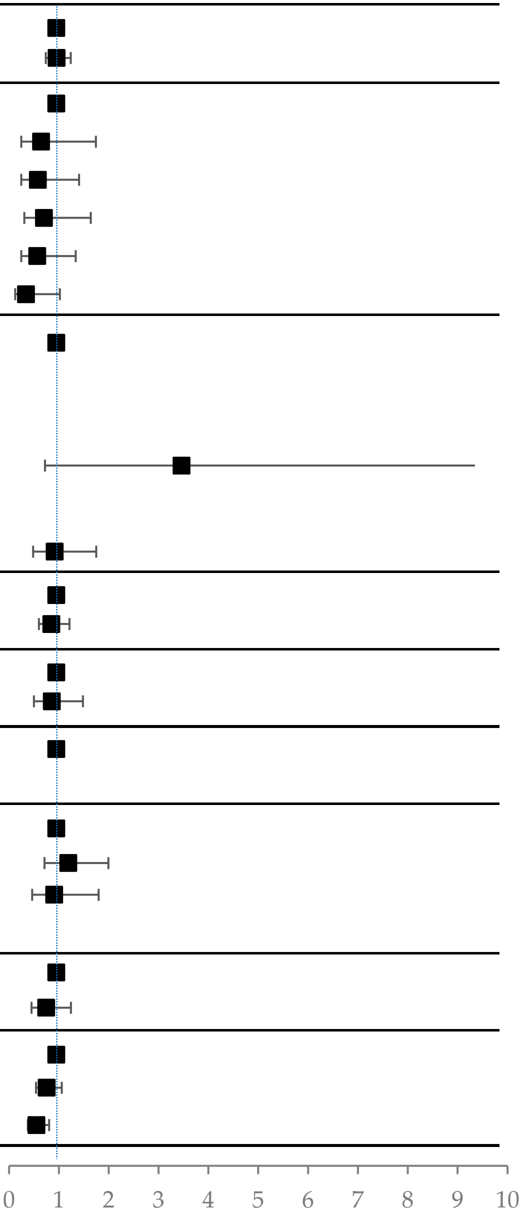

Trait	Group	KRAS G12C	OR	95% CI		p
Primary sidedness	Left colon or rectum (N=2418)	163 (6.74%)	1	ref.		
	Right colon (N=819)	35 (4.27%)	0.618	0.425	0.898	0.012
Primary localization (colon vs rectum)	Colon (N=2060)	107 (5.19%)	1	ref.		
	Rectum (N=1533)	111 (7.24%)	1.425	1.083	1.874	0.011
Sample type	Surgery (N=2292)	140 (6.11%)	1	ref.		
	Endoscopy (N=699)	45 (6.44%)	1.058	0.748	1.497	0.752
	Needle (N=102)	12 (11.76%)	2.05	1.096	3.833	0.025
Tissue source (primary vs metastatic)	Primary (N=2529)	153 (6.05%)	1	ref.		
	Metastatic (N=341)	30 (8.80%)	1.498	0.995	2.255	0.053
Tissue source	Primary (N=2529)	153 (6.05%)	1	ref.		
	Metastatic - other (N=293)	23 (7.85%)	1.323	0.838	2.087	0.229
	Metastatic - locore-gional (N=44)	5 (11.36%)	1.991	0.774	5.124	0.153
	Metastatic - CNS (N=4)	2 (50.00%)	15.529	2.173	110.997	0.006
Biopsy vs surgery for metastatic tissues	Surgery (N=450)	32 (7.11%)	1	ref.		
	Biopsy (N=124)	14 (11.29%)	1.662	0.857	3.224	0.132
Geographic region	Śląskie (N=692)	40 (5.78%)	1	ref.		
	Łódzkie (N=581)	39 (6.71%)	1.173	0.744	1.85	0.493
	Małopolskie (N=436)	36 (8.26%)	1.467	0.919	2.341	0.108
	Dolnośląskie (N=412)	26 (6.31%)	1.098	0.66	1.828	0.719
	Mazowieckie (N=380)	22 (5.79%)	1.002	0.586	1.712	0.995
	Zachodniopomorskie (N=237)	10 (4.22%)	0.718	0.353	1.459	0.36
	Podkarpackie (N=245)	11 (4.49%)	0.766	0.387	1.518	0.445

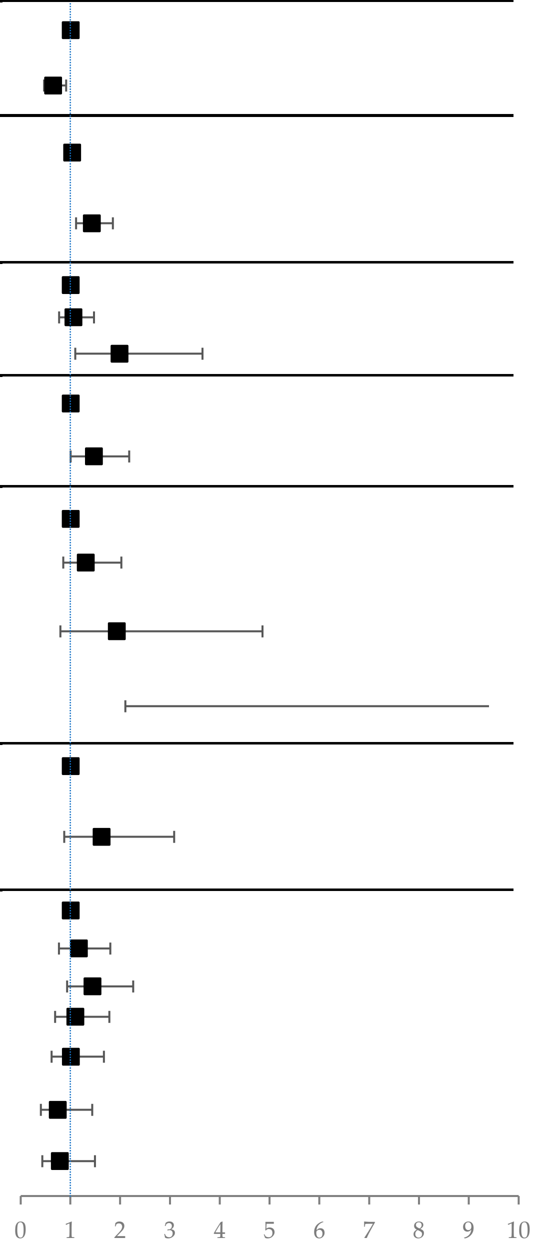

Trait	Group	KRAS G12C	OR	95% CI		p
	Pomorskie (N=200)	10 (5.00%)	0.858	0.421	1.748	0.673
	Wielkopolskie (N=175)	11 (6.29%)	1.093	0.549	2.177	0.8
	Opolskie (N=161)	9 (5.59%)	0.965	0.458	2.032	0.926
	Lubuskie (N=154)	13 (8.44%)	1.503	0.783	2.884	0.221
	Warmińsko-mazur-skie (N=109)	7 (6.42%)	1.119	0.488	2.565	0.791
	Kujawsko-pomorskie (N=21)	3 (14.29%)	2.717	0.768	9.609	0.121
	Lubelskie (N=9)	0 (0.00%)	--	--	--	--
	Podlaskie (N=1)	0 (0.00%)	--	--	--	--

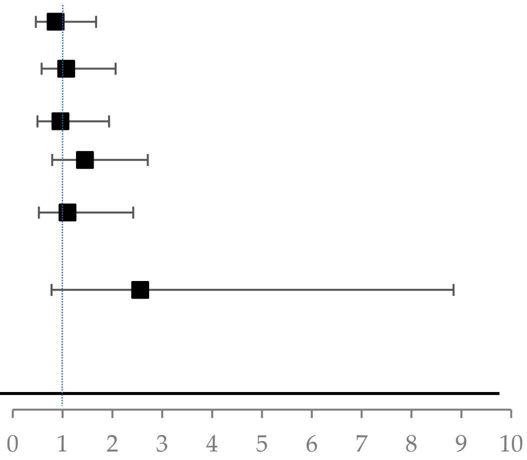

Right colon (proximal) included caecum, ascending colon, hepatic flexure and transverse colon. Left colon (distal) included splenic flexure, descending and sigmoid colon. Rectum included rectosigmoid tumours. MANEC – mixed adeno-neuroendocrine cancer.
p - univariate logistic regressions.
